# Supplementary material for: Collaborative intelligence in AI: Evaluating the performance of a council of AIs on the USMLE
Source: PLOS Digit Health. 2025 Oct 9;4(10):e0000787. doi: 10.1371/journal.pdig.0000787 (PMC12510544; doi:10.1371/journal.pdig.0000787)
Supplement: S2 File — (ZIP) [file pdig.0000787.s002.zip › S2 Data Raw data files containing unprocessed question inputs and LLM outputs including transcripts of deliberations/STEP3_Question-79-1_Type-MC-NJ.docx]

**Facilitator** **[Round 1]:** A 25-year-old man, who was admitted to the hospital 5 hours ago because of nausea, light-headedness, and muscle aches that began after he completed a marathon, now reports worsening pain in his right leg. On admission, the patient reported only mild muscle aching, but he now rates the pain in his right lower extremity as a 9 on a 10-point scale. Laboratory studies on admission were notable for a serum creatine kinase concentration of 10,000 U/L and a serum creatinine concentration of 1.7 mg/dL. Since admission the patient has received 5 L of 0.9% saline and his nausea and light-headedness have resolved. He is alert and fully oriented. Vital signs are temperature 36.9°C (98.5°F), pulse 88/min, respirations 16/min, and blood pressure 126/82 mmHg. Pulse oximetry on room air shows an oxygen saturation of 97%. Cardiopulmonary and abdominal examinations disclose no abnormalities. Passive flexion of the toes elicits pain over the anterior portion of the right lower extremity below the knee. Distal pulses are present in the lower extremities bilaterally. The remainder of the physical examination discloses no abnormalities. Results of laboratory studies obtained 1 hour ago are shown: Serum: Urea nitrogen 12 mg/dL Creatinine 1.2 mg/dL Na+ 140 mEq/L K+ 4.0 mEq/L Cl− 100 mEq/L HCO3 −24 mEq/L Blood: Hemoglobin 14.0 g/dL WBC 14,000/mm3 Neutrophils, segmented 60% Which of the following is the most appropriate next step in management? (A) Application of ice to the right lower extremity (B) Cyclobenzaprine therapy (C) Measurement of lower extremity compartment pressures (D) MRI of the right lower extremity (E) Nonsteroidal anti-inflammatory drug therapy

**AI_A:** RESTATING THE QUESTION: The query inquires about the most fitting next step in the medical management of a man who is experiencing an escalation in leg pain following a marathon. His lab results point to a high concentration of creatine kinase and slightly elevated creatinine, which could indicate an issue related to poor muscle recovery and potential renal damage. The techniques in question involve different modalities of pain relief, physical evaluation, and imaging to identify the root cause of the problem. PLANNING: The assumption seems to be that the patient's pain is primarily due to muscle strain, accounting for the options involving pain relief and muscle relaxants. However, the highly elevated creatine kinase and the location of the pain could hint towards a more severe condition, such as Compartment Syndrome, caused by increased pressure within one of the body's compartments containing muscles and nerves. This condition could be caused by overexertion without proper recovery time (as seen in marathon runners), resulting in muscle swelling and potential nerve damage. CLINICAL REASONING: The major clue pointing towards Compartment Syndrome is the extreme pain the patient experiences when performing passive movement of his toes. This kind of pain is out of proportion for simply post-marathon muscle soreness and given the hyper-elevated creatine kinase level, it seems more likely to result from muscle necrosis due to pressure build-up in a muscular compartment, potentially leading to muscle and nerve damage. CONCLUDING: Keeping the likelihood of Compartment Syndrome in mind, the management should involve confirming the diagnosis by the most expedient method and potentially preparing for necessary intervention to prevent permanent harm. RESPONSE: Therefore, the most appropriate next step in management is to measure lower extremity compartment pressures (Option C) to confirm or rule out Compartment Syndrome.

**AI_D:** RESTATING THE QUESTION: A 25-year-old man started having nausea, light-headedness, and muscle aches after finishing a marathon and got admitted to the hospital around 5 hours ago. The patient's pain in the right leg has worsened since hospital admission, now rated as 9 on a scale of 10. His muscle damage, as was evident from his initial creatine kinase levels and renal function, have improved since admission, with the administration of IV normal saline. Despite this, he continues to experience severe pain in his right lower leg, which is exacerbated with passive toe movements. There are no signs of systemic inflammation or any other abnormal findings on physical examination. The main question here is what the most appropriate next step is in the management of his condition. PLANNING: Given the pattern, there are a couple of relevant assumptions that can be made. One could try to attribute the increased pain to muscle strain from the marathon; however, the severity of the pain suggests it may be something more serious, like compartment syndrome, which occurs due to increased pressure within one of the body's compartments that contains muscles and nerves. This is a potentially dangerous situation, as it can impede blood flow to and from that area, resulting in tissue damage. Another assumption could involve musculoskeletal injury or even deep vein thrombosis, although that would generally come with more specific symptoms, such as localized swelling, heat, and redness. CLINICAL REASONING: The patient's high creatine kinase, intense muscle pain, reported history of strenuous exercise, and the localization and presentation of the pain all point toward a high possibility of compartment syndrome. This can happen as a result of muscle breakdown, leading to an increase in swelling and pressure within a confined space (compartment), thereby compromising blood flow. As this condition can lead to tissue death and severe complications if left untreated, it should be considered a medical emergency. CONCLUDING: Based on these reasons, the risk of compartment syndrome is something we should rule out immediately considering the severity of the patient's pain and exacerbation with movement. RESPONSE: Therefore, the most appropriate next step in management should be (C) Measurement of lower extremity compartment pressures. Once confirmed, the patient would require immediate surgical intervention to prevent potential irreversible muscular and neural damage.

**AI_B:** The presented scenario involves a 25-year-old individual who has developed severe right leg pain after completing a marathon. Initially, symptoms were limited to nausea, light-headedness and muscle aches. Key laboratory findings include an increased creatine kinase concentration, which is an enzyme that can indicate muscle damage, and a serum creatinine concentration indicative of kidney function. To better understand the patient's condition and develop an appropriate management strategy, we will need to evaluate the patient's symptoms, clinical signs and laboratory findings. The assumptions currently are that the patient's pain is due to muscle soreness or injury related to the marathon. However, we should question this assumption. Could the patient's symptoms be a result of a more serious condition like compartment syndrome given the severe pain and his recent rigorous physical activity? Or perhaps there could be a possibility of renal injury due to rhabdomyolysis as the elevated creatine kinase suggests muscle breakdown? The next step in management should focus on determining the cause of the severe pain. In clinical reasoning, taking into account the post-marathon nature of the symptoms, the increased severity of pain in the right leg, and the laboratory findings indicating muscle injury, the suspicion for compartment syndrome is high. This condition occurs when excessive pressure builds within an enclosed muscle space in the body, typically caused by swelling after an injury. In this case, the marathon and the potential dehydration could have led to muscle breakdown, resulting in the swelling and subsequent compartment syndrome. This could also explain why he developed rhabdomyolysis marked by very high serum levels of creatinine and creatine kinase. Rhabdomyolysis occurs when there's a rapid breakdown of muscle that leads to release of intracellular contents, including myoglobin, into the circulation. Myoglobin is harmful to the kidney and can cause kidney damage. In conclusion, the worsening and severe pain in the patient's right leg, along with his recent marathon run, and the elevated creatinine and creatine kinase concentrations suggest the presence of compartment syndrome. The most appropriate next step in the management of this patient should align with confirming this diagnosis and addressing it promptly due to the serious nature of this condition. Therefore, among the presented options, the best next step in management would be (C) Measurement of lower extremity compartment pressures, as this will confirm the diagnosis of compartment syndrome. Rapid confirmation of this could then lead to necessary surgical intervention, namely a fasciotomy, to relieve the increased pressure within the muscle compartment and prevent further complications such as tissue necrosis or renal damage.

**AI_E:** RESTATING THE QUESTION: A man, who recently finished a marathon, is currently admitted to the hospital for symptoms initially mild but have progressively worsened over time, particularly severe pain in his right leg. Upon admission, his laboratory results showed increased creatine kinase and creatinine levels. He's received adequate hydration to alleviate nausea and light-headedness but the leg pain persists. Cardiovascular, pulmonary, and abdominal examinations showed no abnormalities. However, the pain in the right leg is exacerbated with passive toe flexion. Given this situation, what would be the most advantageous next step in managing this patient's condition? PLANNING: The assumptions here could be: a) that the pain is due to muscle overexertion from the marathon, or b) that the patient has developed a more serious condition known as compartment syndrome. We should question these assumptions due to the severity and localization of pain, and the laboratory findings. In the case of simple muscle overexertion, pain is generally diffuse and not localized as the one patient is experiencing. Creatine kinase would be elevated, but creatinine would not be significantly altered, contrarily to what have been found here. Therefore, it may be more plausible to consider compartment syndrome (especially given the man's intense physical activity, elevated creatine kinase, and the specific pain with toe flexion -- specific signs for compartment syndrome), which results from increased pressure within a muscle compartment that can compromise circulation to the area and cause ischemia. CLINICAL REASONING: Now we should consider the step-by-step approach for each assumption. If muscle overexertion were the cause, conservative treatments like rest, ice, NSAIDs, or cyclobenzaprine a muscle relaxant, could manage pain. However, if the compartment syndrome assumption is right, consequences of not treating could be disastrous, possibly leading to muscle and nerve damage and even the need for amputation. Furthermore, elevated creatine kinase and specific pain during passive toe flexion already raise suspicion for compartment syndrome. Thus, to validate this hypothesis, one needs to measure the compartment pressures. CONCLUDING: The step-by-step analysis of each clinical assumption reveals that the most clinically significant approach to take in this case is linked to the worst-case scenario - the possibility of compartment syndrome. While muscle overexertion could explain several of the symptoms, it fails to address the severity of the patient's pain, the rising creatinine levels, and the specific findings on physical examination. The potential for dire consequences in the face of compartment syndrome makes it an urgent clinical priority, hence compartment pressure measurement is necessary to manage this case appropriately. RESPONSE: The most appropriate next step in the management of this patient's condition is to measure lower extremity compartment pressures (Choice C).

**AI_C:** RESTATING THE QUESTION: To summarize, we have a 25-year-old man who was admitted to the hospital due to symptoms of nausea, light-headedness, and muscle aches that started after he completed a marathon. His condition has since escalated, now experiencing severe pain in his right leg. Laboratory studies at the time of admission showed a significant surge in serum creatine kinase, indicative of muscle damage, and slightly elevated serum creatinine suggestive of beginning renal stress. Despite receiving rehydration therapy, the leg pain has intensified. The physical examination is essentially unremarkable except that flexion of the toes of the right leg provokes acute pain. Updated lab results show a decreasing trend in creatinine levels, inferring an improving kidney function, and the elevated WBC count suggests some inflammatory response. The question is about what should be the next step in managing his condition from the given choices. PLANNING: The assumptions deriving from the clinical and lab observations are either the patient has a severe muscle strain or has developed exertional rhabdomyolysis leading to compartment syndrome. The commonly employed tools for management, such as application of ice, anti-inflammatory drugs, muscle relaxants or MRI of the affected site might be beneficial in case of a strain. But if it's a compartment syndrome, i.e., conditions characterized by increased pressure in a muscle compartment that can lead to muscle and nerve damage, a specific procedure of pressure measurement is needed. CLINICAL REASONING: Considering the patient's history of completing a marathon followed by muscle ache, and a significantly elevated creatine kinase level, he likely developed exertional rhabdomyolysis. This occurs when muscle injury results in the release of muscle cell contents, including myoglobin, into the bloodstream. Myoglobin is toxic to renal tubular cells, leading to acute kidney injury. Severe rhabdomyolysis can also cause compartment syndrome due to the inflammation and swelling in the muscles. Given the severity of his leg pain despite rehydration, this is a concern. The options of applying ice, using anti-inflammatory medication, giving muscle relaxants, and doing an MRI may help with muscle strains or minor injuries. However, these wouldn't be sufficient or appropriate if the patient has developed compartment syndrome. CONCLUDING: Compartment syndrome, a potential and serious complication of exertional rhabdomyolysis, can cause irreversible muscle and nerve damage if not caught early. This provides us with a clinical priority to ensure we rule out this condition to prevent severe outcomes for the patient. RESPONSE: Given the patient's symptoms, clinical history and the severity of pain which persisted even after hydration— the most appropriate next step in management should be (C) the measurement of lower extremity compartment pressures to rule out compartment syndrome.

**Facilitator** The consensus among the AI responses is that the most appropriate next step in the management of the patient's condition is to measure lower extremity compartment pressures (Option C). This conclusion is based on the patient's symptoms, clinical history, and laboratory findings. The patient's severe pain in the right leg, recent marathon run, and elevated creatinine and creatine kinase concentrations suggest the presence of compartment syndrome, a condition that occurs when excessive pressure builds within an enclosed muscle space in the body. This condition can lead to muscle and nerve damage if not promptly addressed. Therefore, the measurement of lower extremity compartment pressures is necessary to confirm this diagnosis and prepare for potential surgical intervention to prevent further complications.
